# A network analysis of the Internet Disorder Scale–Short Form (IDS9-SF): A large-scale cross-cultural study in Iran, Pakistan, and Bangladesh

**DOI:** 10.1007/s12144-022-03284-8

**Published:** 2022-06-09

**Authors:** Li Li, Mohammed A. Mamun, Firoj Al-Mamun, Irfan Ullah, Ismail Hosen, Syed Ahsan Zia, Ali Poorebrahim, Morteza Pourgholami, Chung-Ying Lin, Halley M. Pontes, Mark D. Griffiths, Amir H. Pakpour

**Affiliations:** 1 School of Humanities and Social Sciences, Gannan Medical University, Ganzhou, China; 2 CHINTA Research Bangladesh, Savar, Dhaka, Bangladesh; 3 Kabir Medical College, Gandhara University, Peshawar, Pakistan; 4 Karachi Medical and Dental College, Karachi, Pakistan; 5 Guilan University of Medical Sciences, Rasht, Islamic Republic of Iran; 6 Institute of Allied Health Sciences, College of Medicine, National Cheng Kung University, Tainan, 70101 Taiwan; 7 Biostatistics Consulting Center, National Cheng Kung University Hospital, College of Medicine, National Cheng Kung University, Tainan, Taiwan; 8 Department of Occupational Therapy, College of Medicine, National Cheng Kung University, Tainan, Taiwan; 9 Department of Public Health, College of Medicine, National Cheng Kung University, Tainan, Taiwan; 10 Department of Organizational Psychology, Birkbeck, University of London, London, UK; 11 International Gaming Research Unit, Psychology Department, Nottingham Trent University, Nottingham, UK; 12 Social Determinants of Health Research Center, Research Institute for Prevention of Non-Communicable Diseases, Qazvin University of Medical Sciences, Qazvin, Iran; 13 Department of Nursing, School of Health and Welfare, Jönköping University, Barnarpsgatan 39, 55111 Jönköping, Sweden; 14 Department of Public Health, University of South Asia, Dhaka, Bangladesh; 15 Department of Public Health, Daffodil International University, Dhaka, Bangladesh; 16 Department of Public Health and Informatics, Jahangirnagar University, Savar, Dhaka, Bangladesh

**Keywords:** Addictive behavior, Addiction, Cross-country, Internet, Network analysis

## Abstract

The Internet Disorder Scale–Short Form (IDS9-SF) is a validated instrument assessing internet disorder which modified the internet gaming disorder criteria proposed in the fifth edition of the *Diagnostic and Statistical Manual of Mental Disorders* (DSM-5). However, the relationships between the nine items in the IDS9-SF are rarely investigated. The present study used network analysis to investigate the features of the IDS9-SF among three populations in Bangladesh, Iran, and Pakistan. Data were collected (*N* = 1901; 957 [50.3%] females; 666 [35.0%] Pakistani, 533 [28.1%] Bangladesh, and 702 [36.9%] Iranians) using an online survey platform (e.g., *Google Forms*). All the participants completed the IDS9-SF. The central-stability-coefficients of the nine IDS9-SF items were 0.71, 0.89, 0.96, 0.98, 0.98, 1.00, 0.67, 0.79, and 0.91, respectively. The node centrality was stable and interpretable in the network. The Network Comparison Test (NCT) showed that the network structure had no significant differences among Pakistani, Bangladeshi, and Iranian participants (*p*-values = 0.172 to 0.371). Researchers may also use the IDS9-SF to estimate underlying internet addiction for their target participants and further explore and investigate the phenomenon related to internet addiction.

**Supplementary Information:**

The online version contains supplementary material available at 10.1007/s12144-022-03284-8.

## Introduction

Technology use in the present era is an important activity in individuals’ lives. Moreover, internet use has significantly expanded given that hardware and Wi-Fi access have become convenient and affordable. As a result, modern society has integrated internet use into life routines for almost every individual (Lee et al., 2016; Li et al., 2021; Oluwole et al., 2021; Poon et al., 2021). Apart from the many benefits and convenience brought by the internet (e.g., online interaction without the restrictions of distance; online shopping without the restrictions of business hours), there is mounting evidence reporting the adverse effects emerging from excessive internet use, especially among a minority of individuals such as young adults and adolescents (Alimoradi et al., 2019; Kadavala et al., 2021; Ranjan et al., 2021; Patel et al., 2021; Tsai et al., 2018).

In particular, some commercial companies using internet as a platform to sell their products have designed highly interactive features and services (e.g., forums) to attract potential consumers worldwide. Such consumers or users of these internet-related products may therefore have difficulties in controlling their craving for internet use due to the potentially addictive features of these applications (Agbaria, 2020; Gu, 2020; Pontes et al., 2015). Such adverse effects of internet use have been described and summarized by the Interaction of Person-Affect-Cognition-Execution (I-PACE) model (Brand et al., 2016, 2019). This model accounts for the factors comprising the pathology of problematic internet use and consequences of problematic internet use. Therefore, healthcare providers and policymakers should not ignore the adverse effects resulting from harmful internet use.

Although there are numerous activities that can be engaged online via the internet, only online gaming in the form of ‘gaming disorder’ has been recognized as a behavioral addiction in the medical and psychiatry field (Billieux et al., 2021; Kuss et al., 2017). More specifically, gaming disorder has been proposed as a formal diagnosis in the eleventh revision of the *International Classification of Diseases* (ICD-11) and Internet Gaming Disorder (IGD) has been listed as a tentative disorder in the fifth edition of the *Diagnostic and Statistical Manual of Mental Disorders* (DSM-5) within Section III. Indeed, the negative effects of disordered gaming, such as inducing sleep problems, psychological distress, and social interaction impairments (Chen et al., 2020a, 2021a, b, c; Fung et al., 2021; Kwok et al., 2021; Wong et al., 2020), have been extensively investigated and discussed widely in the recent literature. Healthcare professionals have taken an increasing interest and raised their concerns due to the (i) evidence of negative effects resulting from disordered gaming, (ii) inclusion of disordered gaming in diagnostic manuals, and a (iii) relatively high prevalence rate (i.e., ~3% worldwide) as reported in recent systematic reviews and meta-analyses (e.g., Kim et al., 2022; Stevens et al., 2021). However, other negative impacts of different putative online addictions should not be ignored (e.g., social media addictions) (Chen et al., 2020b). Nevertheless, by adapting the IGD criteria proposed by the DSM-5, it is possible to assess internet addiction more generally within a set of pre-existing and well-supported set of clinical criteria (Pontes et al., 2019).

Researchers working in the field of online addictions asserted the importance of having an appropriate and effective assessment tool to evaluate symptoms of internet addiction, and many psychometric assessment tools have been developed over the past two decades (King et al., 2020; Pontes, 2016) ever since the first internet addiction scale (i.e., Internet Addiction Test) was published in 1998 (Young, 1998). However, because disordered gaming is the only online addictive behavior to have been formally recognized as a disorder (American Psychiatric Association, 2013), most psychometric tests assessing internet addiction do not offer a robust and well-defined diagnostic framework (Musetti et al., 2016; Young, 1998). To overcome this issue, the Internet Disorder Scale–Short Form (IDS9-SF) was developed (Pontes & Griffiths, 2016, see the developer’s website for further information: www.halleypontes.com/ids9-sf) based on the APA framework, which is operationalized through the nine criteria proposed in the DSM-5 for IGD (American Psychiatric Association, 2013). More specifically, the wording of the nine IGD criteria was slightly modified with a focus on online leisure activities (i.e., not considering online activities associated with educational or occupational duties) (Pontes & Griffiths, 2016). Therefore, choosing the IDS9-SF for validation is important because it has a theoretical framework that is supported by the DSM-5, includes more contemporary items than other popular instruments (e.g., Internet Addiction Test which is 25 years old and includes outdated items) and underwent a rigorous process in instrument development (Pontes & Griffiths, 2016).

Although the IDS9-SF appears to be an effective psychometric tool in assessing internet addiction, its psychometric properties have been understudied. More specifically, only four studies have examined its psychometric properties in English-speaking, Bangla-speaking, Italian-speaking, and Turkish-speaking populations (Bener et al. 2019; Monacis et al., 2016; Pontes & Griffiths, 2016; Saiful Islam et al., 2020). The psychometric properties of the IDS9-SF have been found to be satisfactory in regards of nomological validity, factorial validity, criterion-related validity, and concurrent validity (Monacis et al., 2016; Pontes & Griffiths, 2016; Saiful Islam et al., 2020).

One important issue in assessing IDS9-SF is in relation to the cultural differences, especially between Western and non-Western countries. More specifically, prior studies using other instruments have found that the prevalence rates were different between Western and non-Western countries. For instance, a higher prevalence of internet use disorder has been reported among individuals in Asian countries (e.g., 51% in Philippines, 48% in Japan, 27% in Bangladesh, 20% in Iran, and 50% in Pakistan) (Ansar et al., 2020; Cheng & Li, 2014; Hassan et al., 2020; Lozano-Blasco et al., 2020; Mak et al., 2014; Modara et al., 2017) than among Western countries (e.g., 2% to 8% among European countries of Germany, Italy, Romania, Estonia and Spain; Cheng & Li, 2014; Lozano-Blasco et al., 2020). Such differences could be attributed to cultural beliefs and modesty standards. For example, Bangladesh, Pakistan, and Iran are collectivist countries and people living in these countries are likely to connect their relationships (Haque & Mohammad, 2013; Nordfjærn & Zavareh, 2016). Therefore, the internet is a good medium for people in these non-Western countries to interact with friends and peers, which for a minority may later develop into an internet use disorder. Consequently, the cultural difference between Western and non-Western countries can be an important factor in the development of internet use disorder.

Apart from the cultural differences between Western and non-Western countries, the relationships between the nine items in the IDS9-SF have not yet been thoroughly investigated. More specifically, prior studies used factor analyses and Pearson correlations to understand how the nine items associated with each other in the IDS9-SF (Monacis et al., 2016; Pontes & Griffiths, 2016; Saiful Islam et al., 2020), and there has been no evidence utilizing network analysis to visualize and portray the relationships between the scale’s nine items.

Network analysis, which is described as having the features of *“structure, positions, and dyadic properties and the overall ‘shape’ of ties on graph-theoretic properties”* (Borgatti et al., 2009, p. 894), has been used in social science research over the past two decades because it can quickly construct a visual model describing relationships between numerous variables (Li et al., 2021). Therefore, network analysis provides the possibility to explore complex psychological mechanisms by examining how variables are inter-related within a network. This approach is especially promising when the psychological mechanisms cannot be converged or do not fit in any latent variable models estimated using structural equation modeling. With information regarding how the variables interact (i.e., the nine IDS9-SF items in the present study), explanations for the symptoms of a condition or social phenomena can be appropriately outlined. Therefore, network analysis approach has been used as one of the statistical methods to investigate psychopathology (McMally, 2021). Additionally, network analysis provides graphical and quantitative method to describe the traits (e.g., the nine IDS9-SF items in the present study) assessed in a tool, which can be easily interpreted and understood (Marcus et al., 2018).

In order to bridge the gap in the literature regarding the specific item features of the IDS9-SF, the present study used network analysis to investigate the IDS9-SF among three populations in Bangladesh, Iran, and Pakistan while conducting gender and country comparisons using the network structure.

## Methods

### Participants and Procedures

The information below describes how data were collected online from 1901 participants (with a mean age of 26.27 years [SD± 8.05] and 50.3% female [n = 957]) from the three countries (Iran, Pakistan, and Bangladesh). Table 1 shows the participants' descriptive characteristics. The inclusion criteria for participating were: (i) voluntary willingness to participate, (ii) having access to the internet, and (iii) and being at least 16 years of age.

#### Pakistan

The data were collected between March 2020 and July 2020 from the Pakistani young adult population (aged 19–24 years) using *Google Forms.* The sample was recruited using a convenience sampling technique. Data were collected by sharing the survey link on different Pakistani social media sites groups such as *WhatsApp, Facebook,* and *Instagram.* Formal ethics approval was granted by the Department of Psychology, University of Sargodha, Sargodha, Pakistan. Informed consent was obtained from all participants. A total of 666 individuals participated in the study with a mean age of 21.77 years (SD ± 2.50) and 49.7% being female.

#### Bangladesh

A cross-sectional survey was conducted among Bangladeshi students, and data were collected through an online data collection tool (i.e., *Google Forms*) between October 7 and December 1, 2020. The data were collected using a convenience sampling method. The survey link was distributed on popular social media sites (e.g., *Facebook, WhatsApp*, etc.) to collect data. Before data collection, informed consent was taken from the participants after describing the purpose of the study. Participation was voluntary, and the security of their data was also ensured. Formal ethics approval was granted from the ethical review committees of the Institute of Allergy and Clinical Immunology of Bangladesh. A total of 533 individuals participated with a mean age of 22.69 years (SD ± 4.58) and 50.6% being female.

#### Iran

Data were collected online using a safe and secure platform during March 2020 to July 2020. Participants were young adults who were studying in five universities in Qazvin, Iran. An introductory SMS with study information and consent was sent to 1000 randomly selected students from five universities in Qazvin. Online informed consent was obtained before study participation. Formal ethical approval was granted by the Ethics Committee of Qazvin University of Medical Sciences. A total of 702 individuals participated with a mean age of 33.25 years (SD ± 8.7) and 50.7% being female.

### Measures

*Internet Disorder Scale–Short Form (IDS9-SF):* The IDS9-SF (Pontes & Griffiths, 2016) was developed according to the nine criteria for IGD proposed in the DSM-5 (American Psychiatric Association, 2013) and represents the short form of the Internet Disorder Scale (IDS-15) (Pontes & Griffiths, 2017). More specifically, Pontes and Griffiths (2016) modified the wording to make the descriptions in the IDS9-SF focus on internet use in the context of online leisure activities. A five-point Likert scale (1 = Never to 5 = Very often) was used for the IDS9-SF and a higher score indicates a greater level of internet addiction. More specifically, there are nine items in the IDS9-SF and the total score range for the scale is between 9 and 45. Pontes and Griffiths (2016) additionally proposed that five or more of the nine items rating on “very often” can be tentatively used for a strict diagnosis on internet addiction, although they discourage this type of practice.

### Statistical Analysis

Descriptive statistics and network analysis were performed using SPSS 25 (IBM Corp., 2017) and JASP (Jeffrey’s Amazing Statistics Program), respectively. For descriptive statistics, the participants were classified as being at risk of internet addiction or non-disordered internet users based on the criteria suggested by Pontes and Griffiths (2016) for the IDS (i.e., five or more of the nine items were answered “*very often*” [from “1 = *never*” to “5 = *very often*”]). Moreover, a *χ*^2^ test was used to examine whether the proportions of being at risk of internet addiction were different between genders; analysis of variance (ANOVA) with Bonferroni correction was used to examine whether the IDS9-SF scores were different among the three country groups. The R package was used to conduct the Network Comparison Test (NCT) on gender and countries (van Borkulo, 2016). The EBICglasso model was used to estimate network structure and strength, utilizing the Extended Bayesian Information Criterion (EBIC; Chen & Chen, 2008) for a least absolute shrinkage and selection operator (LASSO; Friedman et al., 2008). EBICglasso selected the tuning parameter of 0.5 for a higher specific and sensitive network. Nodes and edges represent study variables and correlations between two nodes, respectively, which together form the network system. Betweenness (degree of connectivity), closeness (the distance centrality), and strength (degree centrality) as the index of the centrality of codes were calculated (Epskamp et al., 2012). The correlation stability coefficient (CS-coefficient) was calculated and at least 0.25 and more than 0.5 is preferable, which indicates a higher node centrality stability (Epskamp et al., 2018). Positive and negative partial correlations are displayed through blue and orange lines, respectively. Stronger relationships between variables are displayed by more saturated and thicker edges. Bootstrapping (1000 times) with 95% confidence intervals was used to estimate edge stability. The NCT was conducted for comparing network structure and global network strengths between gender as well as three different countries.

## Results

### Descriptive Statistics

Less than 5% of the participants (n = 58; 3.1%) were classified as being at risk of internet addiction. Among them, 14 were males, 43 were females [4.5% of 957 females], and one participant [out of 16] who preferred not to report their gender). Moreover, 1843 participants were classed as being non-disordered internet users (96.9%). There was a significant gender difference (*χ*^2^ = 69.57, *p* < 0.001) with females being more likely to be classified as addicted to the internet. The total internet addiction scores were also significantly different among country groups (*F* = 215.618, *p* < 0.001). Bonferroni correction for multiple comparison further demonstrated that there were significant gender difference and country difference for internet addiction. Females had higher internet addiction total score than males and those who preferred not to say. There was a lower internet addiction total score among Iranian participants than among Bangladeshi and Pakistani participants.

### EBICglasso Network Analysis for the Total Sample

**Fig. 1:**
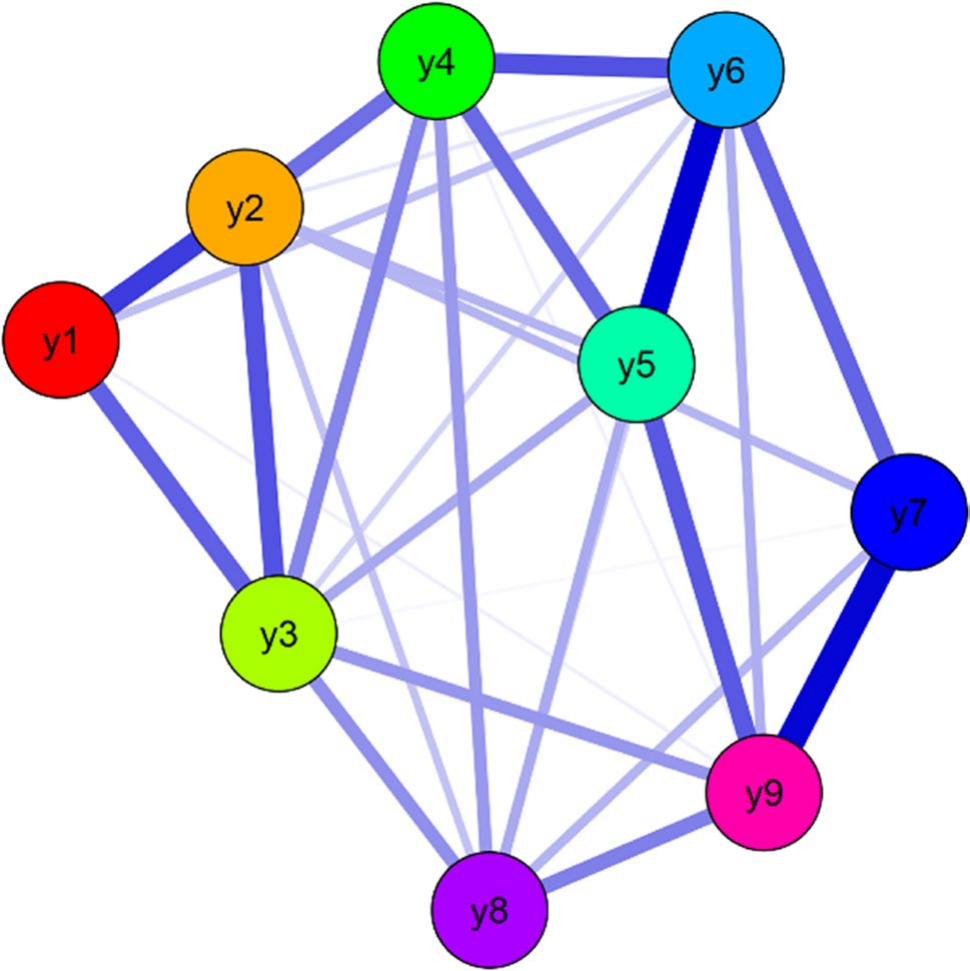
EBICglasso model based on network analysis according to the Internet Addiction scale among 1901 participants. Note: y1-y9 = internet addiction criteria

The EBICglasso network including the nine internet addiction items for the total sample are shown in Fig. 1. Nodes y5 (i.e., *“Have you lost interest in previous hobbies and other leisure activities as a result of being online?*”) and y6 (i.e., *“Have you continued to go online despite knowing it was causing problems between you and other people?*”) had the strongest edge intensity (*r* = 0.306). Nodes y7 (i.e., *“Have you deceived any of your family members, therapists or other people because the amount of time you spend online?*”) and y9 (i.e., *“Have you jeopardized or lost an important relationship, career or an educational opportunity because of your online usage?*”) had also stronger edge intensity (*r* = 0.3) (see Appendix S5). The indices of the nodes are described by betweenness, closeness, and strength (degree) for the total sample are shown in Appendix S6. Node y6 (i.e., *“Have you continued to go online despite knowing it was causing problems between you and other people?*”) was the highest central strength (1.102). The central-stability-coefficients of the nine IDS9-SF items were 0.71, 0.89, 0.96, 0.98, 0.98, 1.00, 0.67, 0.79, and 0.91, respectively (see Appendix S9). The node centrality was stable and interpretable in the network.

### EBICglasso Network Analysis for Males and Females

**Fig. 2:**
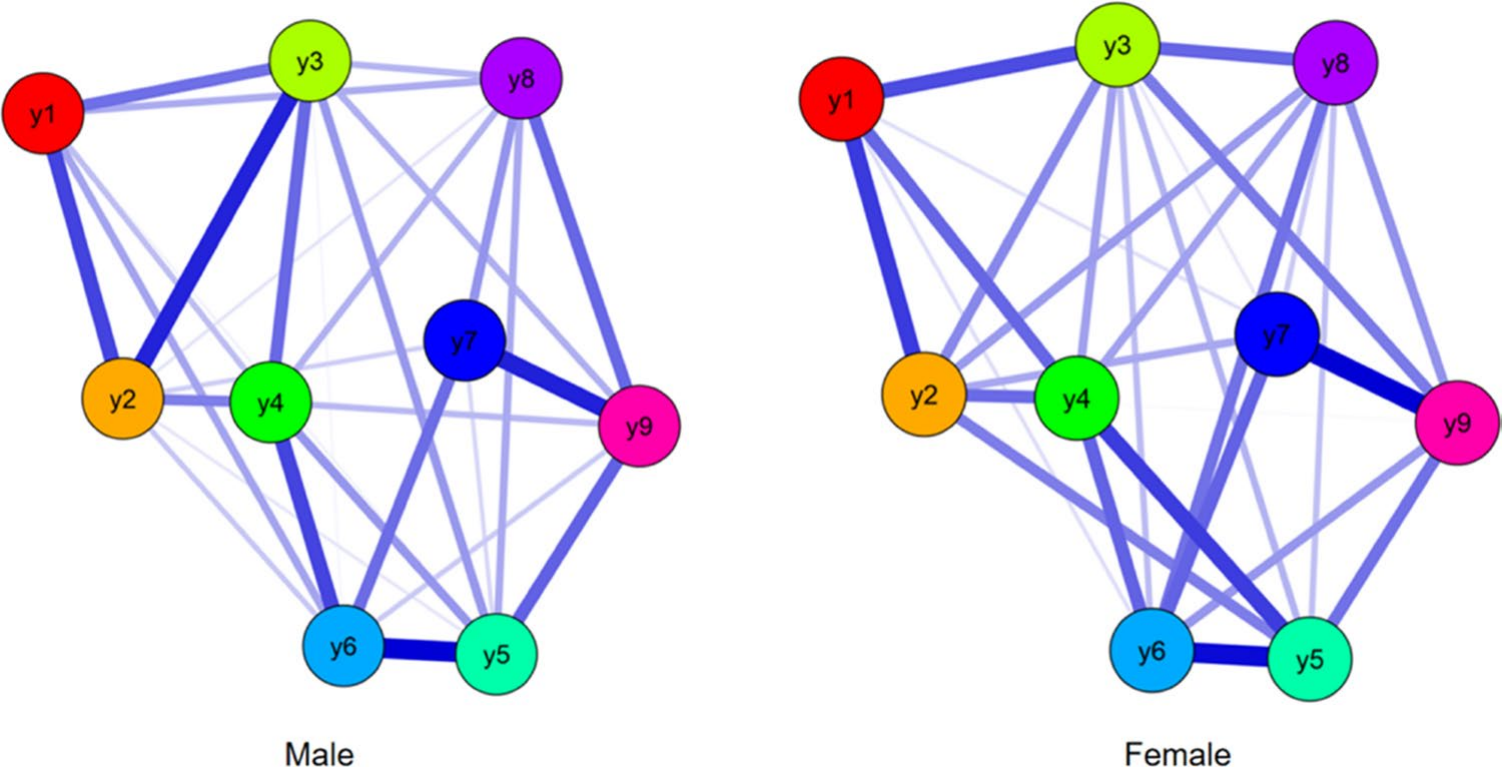
EBICglasso model based on network analysis according to the Internet Addiction scale between gender. Note: y1-y9 = internet addiction criteria

**Fig. 3:**
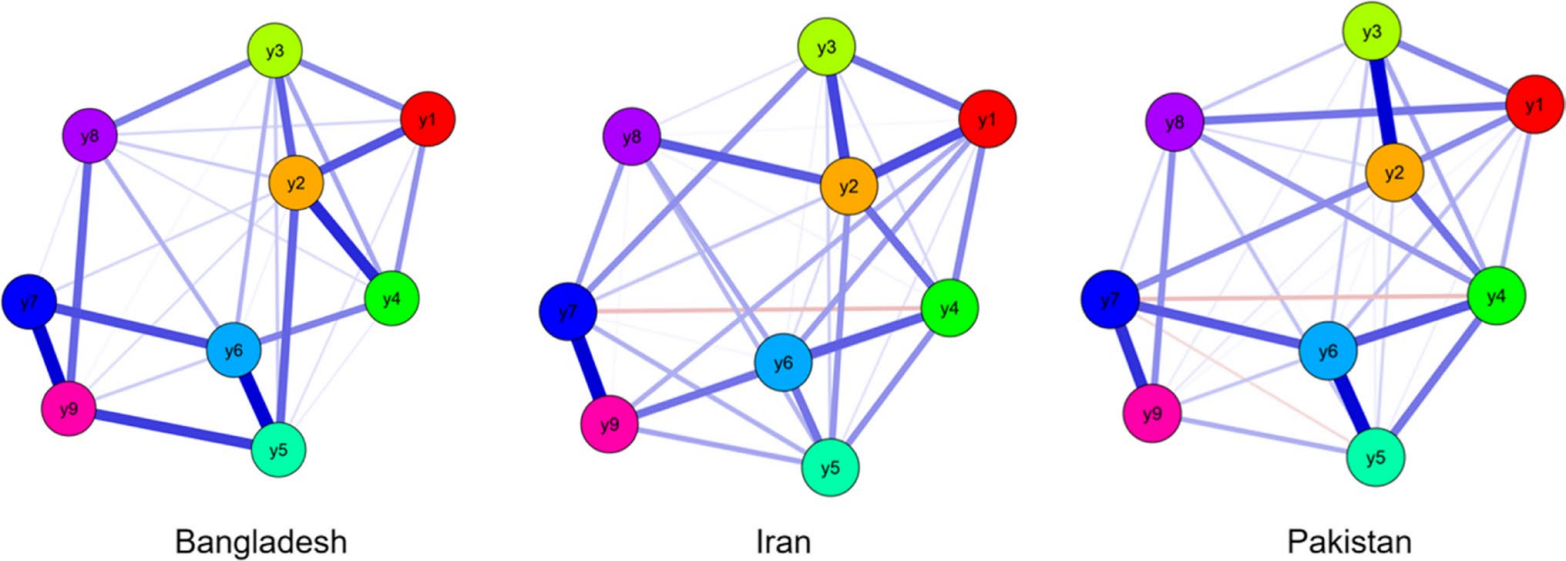
EBICglasso model based on network analysis according to the Internet Addiction scale among 533 Bangladeshi participants (A), 702 Iranian participants (B) and 666 Pakistani participants. Note: y1-y9 = internet addiction criteria

There were 31 non-zero edges in the male network and 29 non-zero edges in the female network (see Figs. 2, 3). Edge weight matrix among males and females are shown in Appendices S10 and S11. The edge of y5 and y6 had the strongest intensity among males (*r* = 0.32), while the edge of y7 and y9 had the strongest intensity among females (*r* = 0.296). Standardized estimates of node strength centrality among male and female groups are shown in Appendix S12. The CS-coefficients of the nine items among males and females were 0.72 and 0.69 (y1), 0.86 and 0.91 (y2), 0.97 and 0.97 (y3), 0.97 and 0.97 (y4), 0.97 and 0.98 (y5), 0.99 and 1.00 (y6), 0.7 and 0.66 (y7), 0.75 and 0.82 (y8), and 0.91 and 0.9 (y9), respectively (see Appendices S16 and S17).

**Table 1:** Descriptive characteristics of the sample (n = 1902)

Variables	N (100%)/Mean (±SD)	Internet addiction total score (M ± SD)
Age	26.3 (±8.1)	19.56 (±7.68)
Gender		
Male	928 (48.8%)	18.90 (±7.20)
Female	957 (50.4%)	20.22 (±8.06)
Prefer not to say	16 (0.8%)	18.81 (±8.74)
Country		
Bangladesh	534 (28.1%)	22.67 (±7.71)
Iran	702 (36.9%)	15.28 (±5.63)
Pakistan	666 (35%)	21.58 (±7.51)

### Comparison of Network between Males and Females

Excluding the small sample who preferred not to report their gender (*N* = 16), the NCT showed the network structure was significantly different between males and females (*M* = 0.160, *p* = .049). However, there was no significant differences between genders in relation to the global edge strengths (males = 3.75 vs. females = 3.84, *p* = .116).

### Comparison of Network among Bangladeshi, Iranian, and Pakistani Participants

The NCT showed that the network structure had no significant differences between the Bangladeshi and Iranian participants (*M* = 0.177, *p* = 0.178), the Bangladeshi and Pakistani participants (*M* = 0.144, *p* = 0.371), or the Iranian and Pakistani participants (*M* = .169, *p* = 0.172). In addition, there were no significant differences in global network strengths between the Bangladeshi and Pakistani participants (3.76 vs. 3.75, *p* = 0.924). However, there were significant differences in the global network strengths between the Bangladeshi and Iranian participants (3.76 vs. 3.24, *p* < 0.001), and the Iranian and Pakistani participants (3.24 vs. 3.75, *p* < 0.001).

## Discussion

The present study provides further psychometric evidence concerning the IDS9-SF by adopting a novel psychometric approach. The IDS9-SF is an instrument assessing generalized internet addiction based on the nine criteria in the DSM-5 (American Psychiatric Association, 2013; Pontes & Griffiths, 2016). To date, little psychometric evidence exists concerning the IDS9-SF even though it is a diagnostic-based instrument to help mental health professionals and researchers understand the issue of internet addiction. Nevertheless, the current psychometric evidence on the IDS9-SF as published in the literature is satisfactory according to four empirical studies reporting nomological validity, factorial validity, criterion-related validity, and concurrent validity findings (Bener et al., 2019; Monacis et al., 2016; Pontes & Griffiths, 2016; Saiful Islam et al., 2020). More specifically, the present psychometric findings were evidenced on a relatively diverse sample in that the present sample included three Asian ethnic populations (Bangladeshi, Iranian, and Pakistani) across different socioeconomic levels, Asian ethnic, sex, age, religions, and cultures. However, given that the present sample was recruited using online survey utilizing a convenience sampling method, individuals without internet access or technology literacy were unlikely to participate. Moreover, the present psychometric evidence is especially relevant to Asian countries with similar cultures of Bangladesh, Iran, and Pakistan but not that relevant to Western countries.

Moreover, the unidimensional factor structure of the IDS9-SF has been previously supported by studies employing factor analytic approaches (e.g., exploratory factor analysis and confirmatory factor analysis) (Monacis et al., 2016; Pontes & Griffiths, 2016; Saiful Islam et al., 2020). The present study, with the use of network analysis, strengthens the evidence supporting a unidimensional factor structure for the IDS9-SF (see Fig. 1). However, the unidimensional factor structure appears to have different degrees of associations between the nine IDS9-SF items across genders (male and female) and countries (Bangladesh, Iran, and Pakistan).

To the best of the authors’ knowledge, the IDS9-SF has never been examined in relation to its equivalence across different genders or different ethnic groups. The network analysis results clearly indicate that the IDS9-SF presents with a unidimensional factor structure that is equivalent across male and female, and across Bangladesh, Iran, and Pakistan participants. There is no prior existing evidence on the IDS9-SF regarding its factor structure across different subgroups. However, a similar instrument focusing exclusively on disordered gaming (i.e., Internet Gaming Disorder Scale–Short-Form; IGDS9-SF, Pontes & Griffiths, 2015) shows that the nine DSM-5 criteria for IGD are embedded in the same construct across genders and different ethnic groups (see de Palo et al., 2018; Pontes et al., 2017; Poon et al., 2021; Stavropoulos et al., 2018). Given that both the IDS9-SF and the IGDS9-SF are rooted in the same diagnostic framework (i.e., DSM-5 [APA, 2013]), it is reasonable to postulate that both instruments should share the same unidimensional factor structure with similar psychometric properties. That is, the unidimensional factor structure of the IDS9-SF across different subgroups is supported, similarly to what has been reported for the IGDS9-SF.

The present network analysis further showed that females had a higher rate of internet addiction than males. The findings are aligned with some prior literature showing that females as compared with males had significantly higher level of internet addiction (Chiu et al., 2013; Ha & Hwang, 2014; Yen et al., 2009). A possible reason for the higher level of internet addiction in females than males could be explained by the I-PACE model (Brand et al., 2016). More specifically, the I-PACE indicates that problematic internet use is attributed by the inappropriate coping behaviors for psychological distress (e.g., depression). Given that females as compared with males are more likely to have mood difficulties (Parker & Brotchie, 2010), females as compared with males are more likely to use internet to cope with their mood difficulties. As a result, females had a higher risk than males to experience internet addiction. Another potential plausible explanation for this finding is that the IDS9-SF does not assess application-specific internet use, as such, given that most of the participants recruited to this study were sampled from social media platforms, it is likely that this has contributed to higher symptomatology among females since prevalence rates for social media addiction (a subtype of internet addiction) are typically higher amongst females in comparison to males (see the review by Su et al., 2020) as they have been found to use social media more intensively than their male counterparts (Bányai et al., 2017).

There are some potential limitations in the present study. First, the IDS9-SF has the common problems associated with self-report data, such as memory recall bias and social desirability bias. Therefore, there may be some inaccurate answers when an individual responds to items on the IDS9-SF. However, given that the objective measures of internet addiction (e.g., brain images) are not yet mature, using the IDS9-SF to assess internet addiction seems to be acceptable. Moreover, the use of the IDS9-SF has the added benefit of being a cost-effective tool. Second, the present study did not adopt any external criterion instruments (e.g., IGDS9-SF; Poon et al., 2021) to investigate how internet addiction associates with other behavioral addictions, as previously shown (Rozgonjuk et al., 2021). Therefore, evidence on the concurrent validity of the IDS9-SF was not reported in the present study. Third, the present study recruited participants from Bangladesh, Iran, and Pakistan; therefore, all the participants were Asians. Therefore, the network analysis findings reported cannot be generalized to citizens or residents of any Western countries. Fourth, due to the nature of the IDS9-SF (i.e., non-specific internet addiction assessment), it is important that future research evaluate symptoms of other co-occurring online addictions while assessing generalized internet addiction symptoms so that addition relevant information can be derived in terms of the context of internet use. Lastly, the data collection was performed using an online survey. This was a necessity during the COVID-19 pandemic because it minimized in-person contacts. However, such data collection suffers from the risk of bias sampling (e.g., those who did not have internet access could not participate) and data quality (i.e., one cannot ensure the participants are paying attention or concentrating on the questions in the online survey).

In conclusion, the IDS9-SF can help mental health professionals screen individuals who are at risk of generalized internet addiction. Furthermore, researchers may also use the IDS9-SF to estimate the underlying internet addiction for their target participants and further explore and investigate the phenomenon related to internet addiction in large-scale epidemiological research as is typically done (Pontes and Macur, 2021). Additionally, the IDS9-SF should be used with caution to compare the levels of internet addiction between genders or between country residents given that significant differences were found in the network analysis regarding the associations between the nine items across these subgroups.

## Supplementary information

ESM 1 (DOC 1546 kb)

## Data Availability

The datasets generated for this study are available on reasonable request to the corresponding author.
