# Supplementary material for: A network analysis of the Internet Disorder Scale–Short Form (IDS9-SF): A large-scale cross-cultural study in Iran, Pakistan, and Bangladesh
Source: Curr Psychol. 2022 Jun 9:1–10. Online ahead of print. doi: 10.1007/s12144-022-03284-8 (PMC9177408; doi:10.1007/s12144-022-03284-8)
Supplement: Supplementary file 1 — (DOC 1546 kb) [file 12144_2022_3284_MOESM1_ESM.doc]

**Supplementary materials**


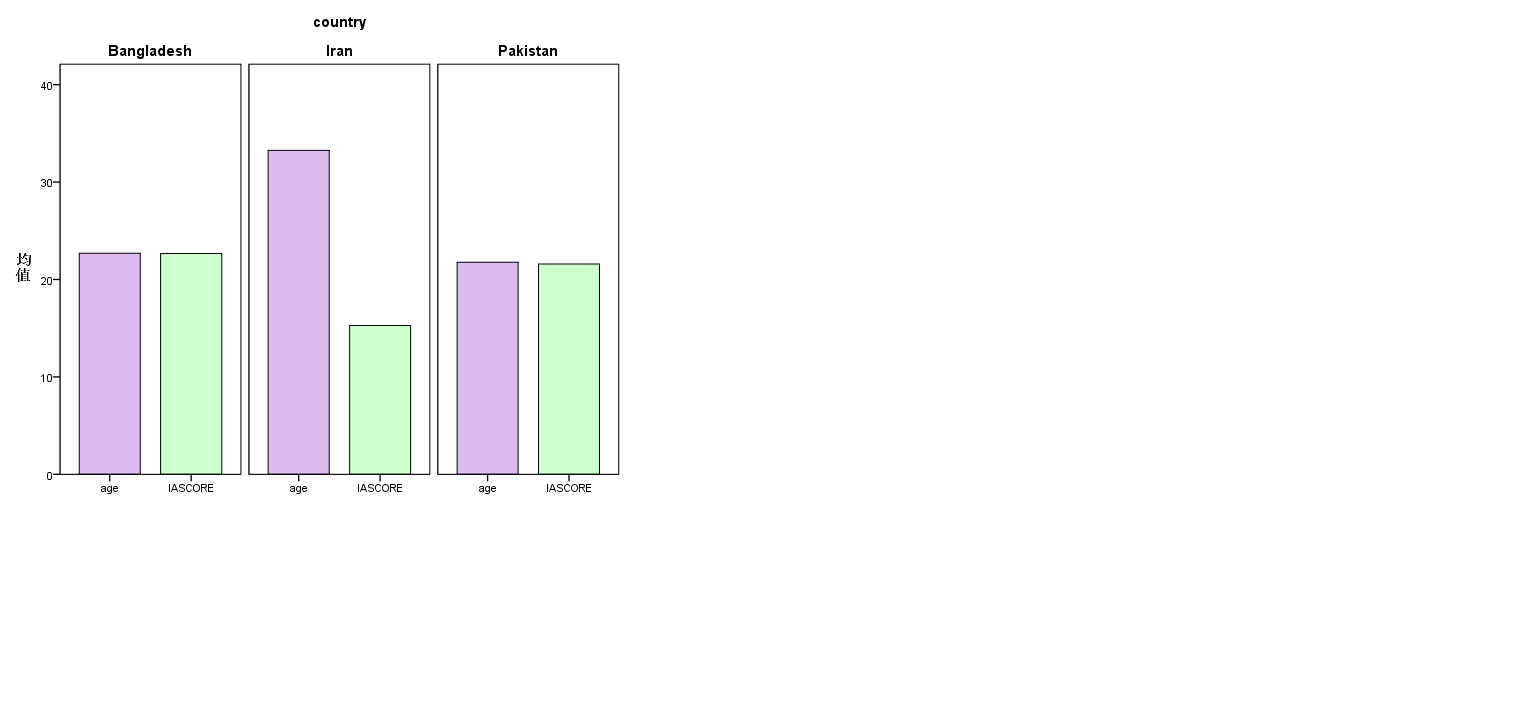


**Appendix S1** Simple bar graph on age and total score of internet addiction (IASCORE) among three countries

**Appendix S2 Edge weight matrix of the facet-level network among 1901 participants**

| Variables | y1 | y2 | y3 | y4 | y5 | y6 | y7 | y8 | y9 |
| --- | --- | --- | --- | --- | --- | --- | --- | --- | --- |
| y1 | 0.000 | 0.234 | 0.190 | 0.137 | 0.000 | 0.077 | 0.000 | 0.051 | 0.019 |
| y2 | 0.234 | 0.000 | 0.207 | 0.172 | 0.090 | 0.030 | 0.088 | 0.073 | -5.100e -6 |
| y3 | 0.190 | 0.207 | 0.000 | 0.145 | 0.103 | 0.053 | 0.009 | 0.134 | 0.125 |
| y4 | 0.137 | 0.172 | 0.145 | 0.000 | 0.178 | 0.208 | 0.000 | 0.122 | 0.016 |
| y5 | 0.000 | 0.090 | 0.103 | 0.178 | 0.000 | 0.306 | 0.000 | 0.101 | 0.199 |
| y6 | 0.077 | 0.030 | 0.053 | 0.208 | **0.306** | 0.000 | 0.186 | 0.062 | 0.097 |
| y7 | 0.000 | 0.088 | 0.009 | 0.000 | 0.000 | 0.186 | 0.000 | 0.090 | 0.300 |
| y8 | 0.051 | 0.073 | 0.134 | 0.122 | 0.101 | 0.062 | 0.090 | 0.000 | 0.152 |
| y9 | 0.019 | -5.100e -6 | 0.125 | 0.016 | 0.199 | 0.097 | **0.300** | 0.152 | 0.000 |

Appendix S3 Centrality measures per variable of the network among 1901 participants

| Variables | Betweenness | Closeness | Strength |
| --- | --- | --- | --- |
| y1 | -0.958 | -1.501 | -1.352 |
| y2 | -0.419 | -0.313 | 0.125 |
| y3 | 1.197 | 0.759 | 0.680 |
| y4 | 1.736 | 1.743 | 0.790 |
| y5 | 0.120 | 0.625 | 0.781 |
| y6 | -0.419 | 0.059 | 1.102 |
| y7 | -0.958 | -0.587 | -1.627 |
| y8 | -0.958 | -1.095 | -0.736 |
| y9 | 0.659 | 0.309 | 0.237 |


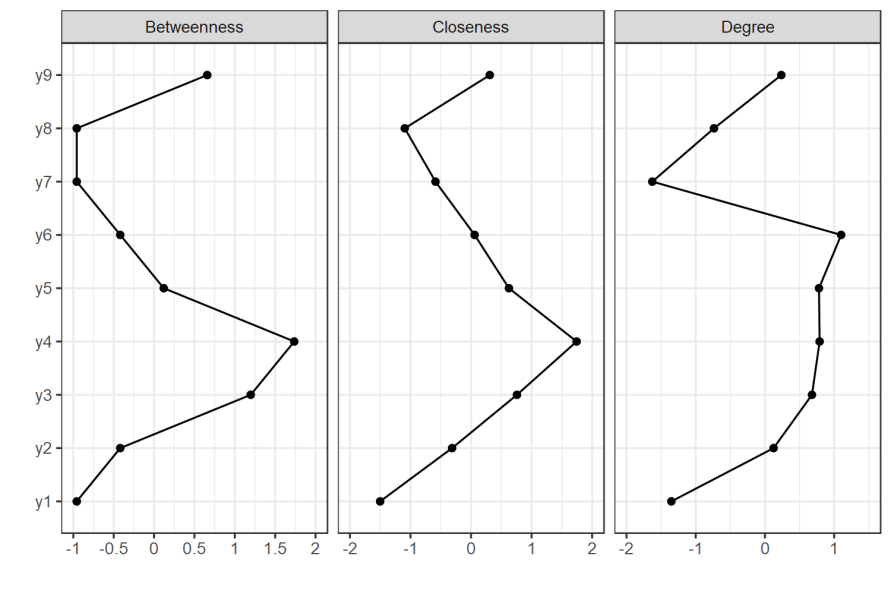


**Appendix S4.** Standardized estimates of node centrality in the network among 1901 participants


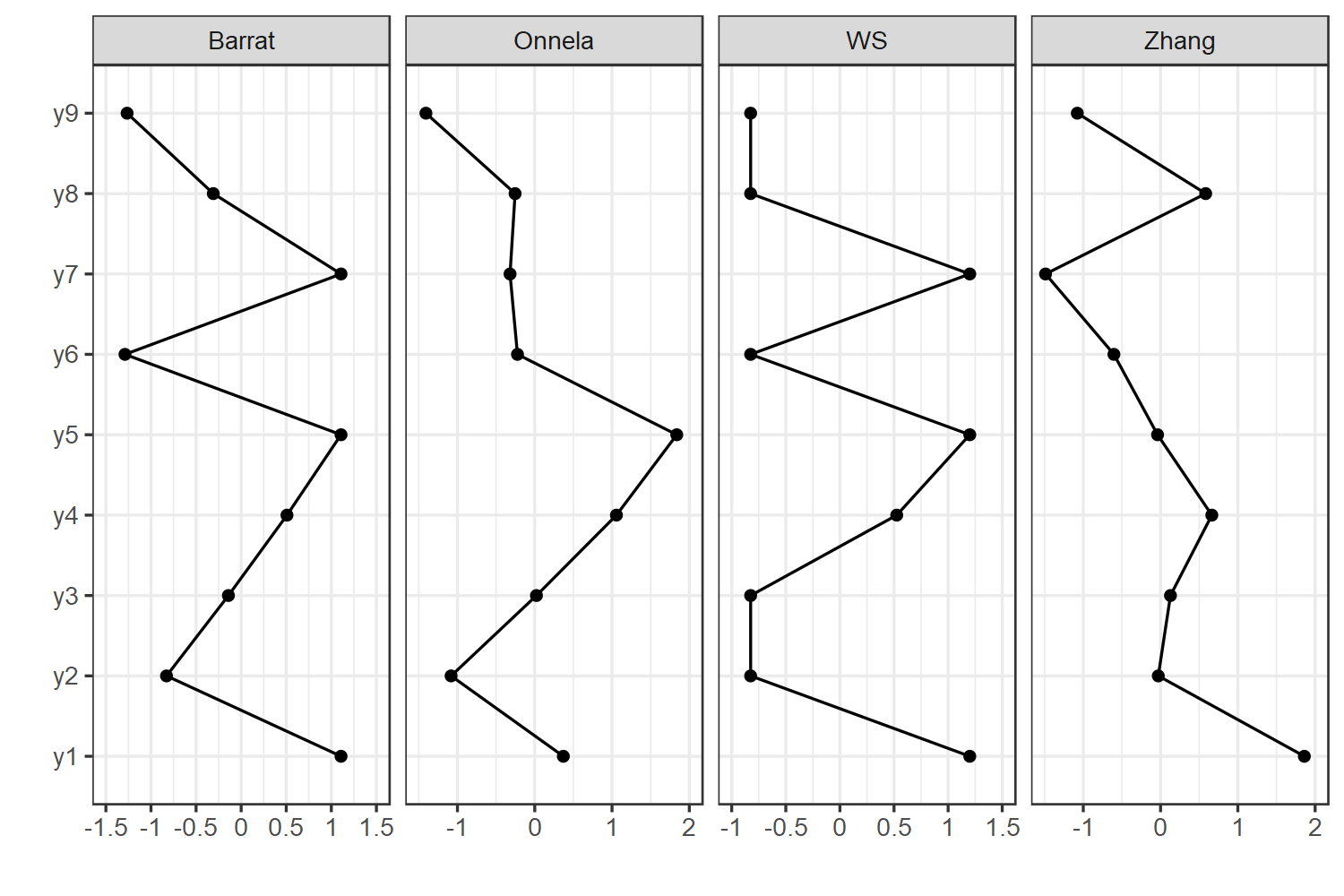


**Appendix S5.** Standardized estimates of node clustering in the network among 1901 participants


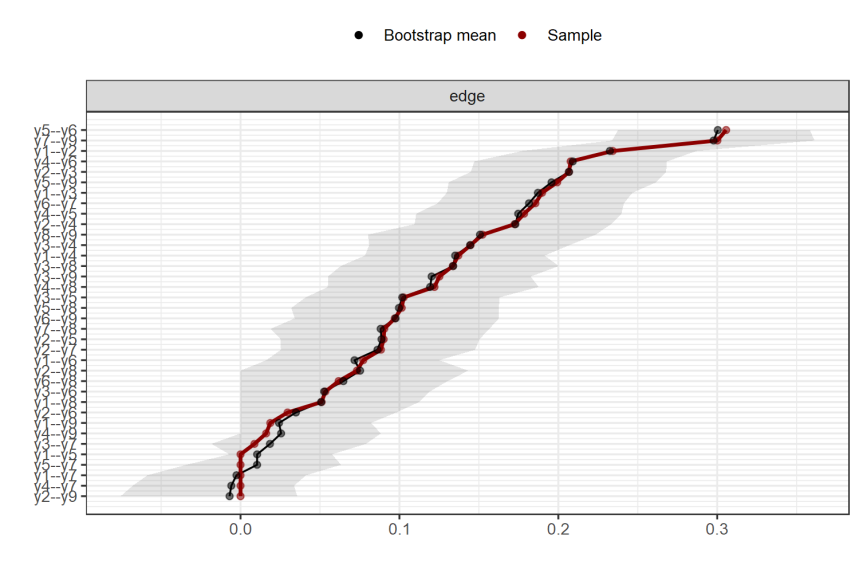


**Appendix S6.** Edge stability in the network among 1901 participants


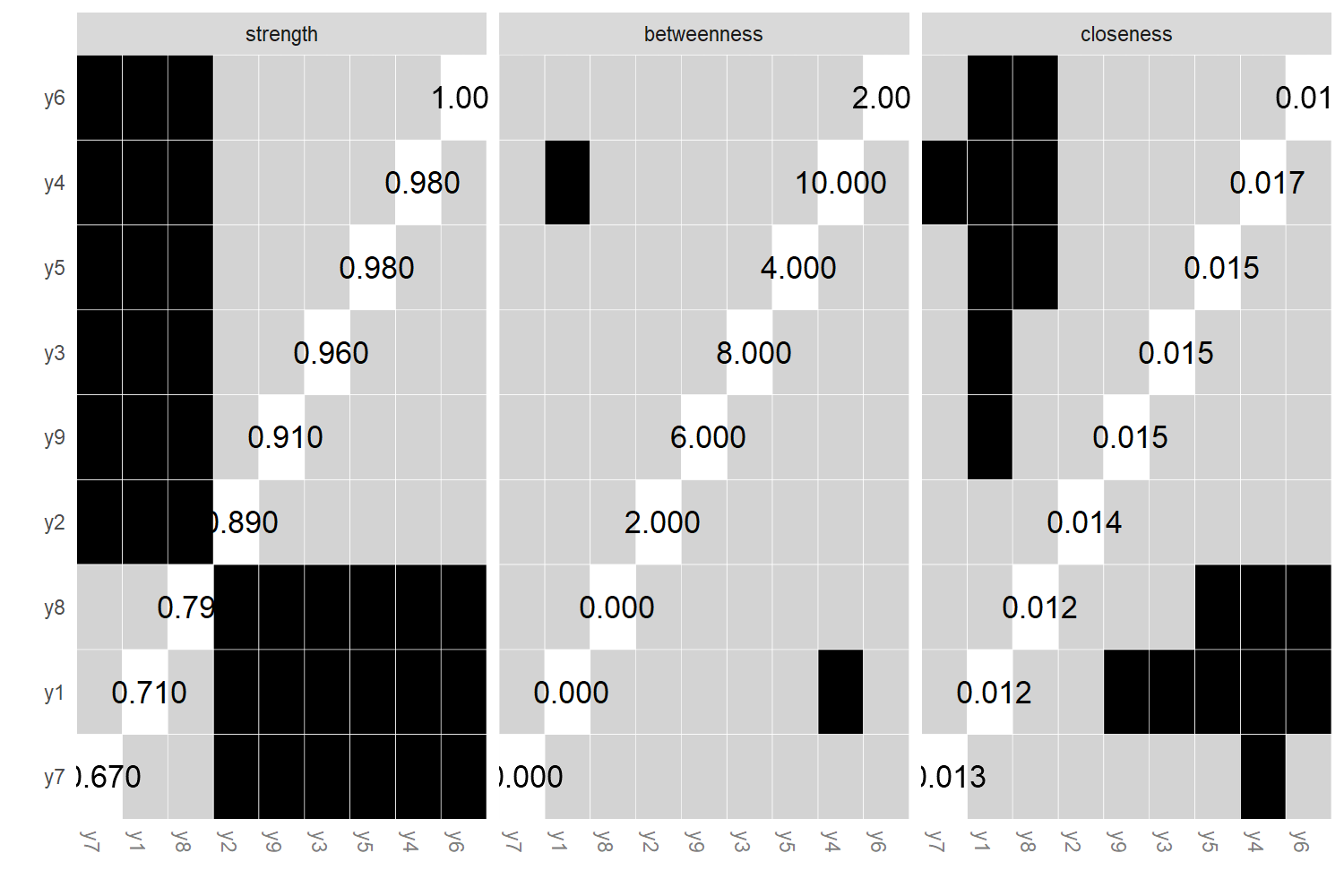


**Appendix S7.** Centrality stability in the network among 1901 participants

**Appendix S8 Edge weight matrix of the facet-level network among 928 males**

| Variables | y1 | y2 | y3 | y4 | y5 | y6 | y7 | y8 | y9 |
| --- | --- | --- | --- | --- | --- | --- | --- | --- | --- |
| y1 | 0.000 | 0.233 | 0.178 | 0.080 | 0.016 | 0.112 | 0.000 | 0.102 | 2.554e -4 |
| y2 | 0.233 | 0.000 | 0.274 | 0.160 | 0.030 | 0.068 | 0.060 | 0.032 | 0.000 |
| y3 | 0.178 | 0.274 | 0.000 | 0.186 | 0.128 | 0.011 | 0.000 | 0.091 | 0.098 |
| y4 | 0.080 | 0.160 | 0.186 | 0.000 | 0.130 | 0.232 | 0.000 | 0.095 | 0.087 |
| y5 | 0.016 | 0.030 | 0.128 | 0.130 | 0.000 | 0.318 | 0.049 | 0.106 | 0.196 |
| y6 | 0.112 | 0.068 | 0.011 | 0.232 | **0.318** | 0.000 | 0.181 | 0.000 | 0.065 |
| y7 | 0.000 | 0.060 | 0.000 | 0.000 | 0.049 | 0.181 | 0.000 | 0.132 | 0.277 |
| y8 | 0.102 | 0.032 | 0.091 | 0.095 | 0.106 | 0.000 | 0.132 | 0.000 | 0.189 |
| y9 | 2.554e -4 | 0.000 | 0.098 | 0.087 | 0.196 | 0.065 | **0.277** | 0.189 | 0.000 |

**Appendix S9 Edge weight matrix of the facet-level network among 957 females**

| Variables | y1 | y2 | y3 | y4 | y5 | y6 | y7 | y8 | y9 |
| --- | --- | --- | --- | --- | --- | --- | --- | --- | --- |
| y1 | 0.000 | 0.228 | 0.207 | 0.176 | 0.000 | 0.037 | 0.000 | 0.000 | 0.036 |
| y2 | 0.228 | 0.000 | 0.137 | 0.172 | 0.150 | 0.000 | 0.100 | 0.116 | 0.006 |
| y3 | 0.207 | 0.137 | 0.000 | 0.109 | 0.087 | 0.076 | 0.027 | 0.178 | 0.153 |
| y4 | 0.176 | 0.172 | 0.109 | 0.000 | 0.225 | 0.182 | 0.000 | 0.108 | 0.000 |
| y5 | 0.000 | 0.150 | 0.087 | 0.225 | 0.000 | 0.285 | 0.000 | 0.072 | 0.164 |
| y6 | 0.037 | 0.000 | 0.076 | 0.182 | **0.285** | 0.000 | 0.181 | 0.164 | 0.121 |
| y7 | 0.000 | 0.100 | 0.027 | 0.000 | 0.000 | 0.181 | 0.000 | 0.060 | 0.296 |
| y8 | 0.000 | 0.116 | 0.178 | 0.108 | 0.072 | 0.164 | 0.060 | 0.000 | 0.126 |
| y9 | 0.036 | 0.006 | 0.153 | 0.000 | 0.164 | 0.121 | **0.296** | 0.126 | 0.000 |

Appendix S10 Centrality measures per variable of the network between gender

| Variables | Males | | | Females | | |
| --- | --- | --- | --- | --- | --- | --- |
|  | Betweenness | Closeness | Strength | Betweenness | Closeness | Strength |
| y1 | -0.504 | -1.018 | -1.263 | -1.376 | -1.376 | -1.480 |
| y2 | -0.504 | -1.010 | -0.108 | -0.491 | -0.491 | 0.177 |
| y3 | 0.063 | 0.760 | 0.815 | 0.393 | 0.393 | 0.670 |
| y4 | 0.630 | 0.839 | 0.838 | 0.393 | 0.393 | 0.654 |
| y5 | 0.063 | 1.003 | 0.859 | 1.278 | 1.278 | 0.733 |
| y6 | 2.331 | 1.402 | 0.993 | 1.278 | 1.278 | 1.203 |
| y7 | -1.071 | -0.885 | -1.449 | -1.376 | -1.376 | -1.641 |
| y8 | -0.504 | -0.939 | -1.039 | -0.491 | -0.491 | -0.453 |
| y9 | -0.504 | -0.153 | 0.352 | 0.393 | 0.393 | 0.137 |


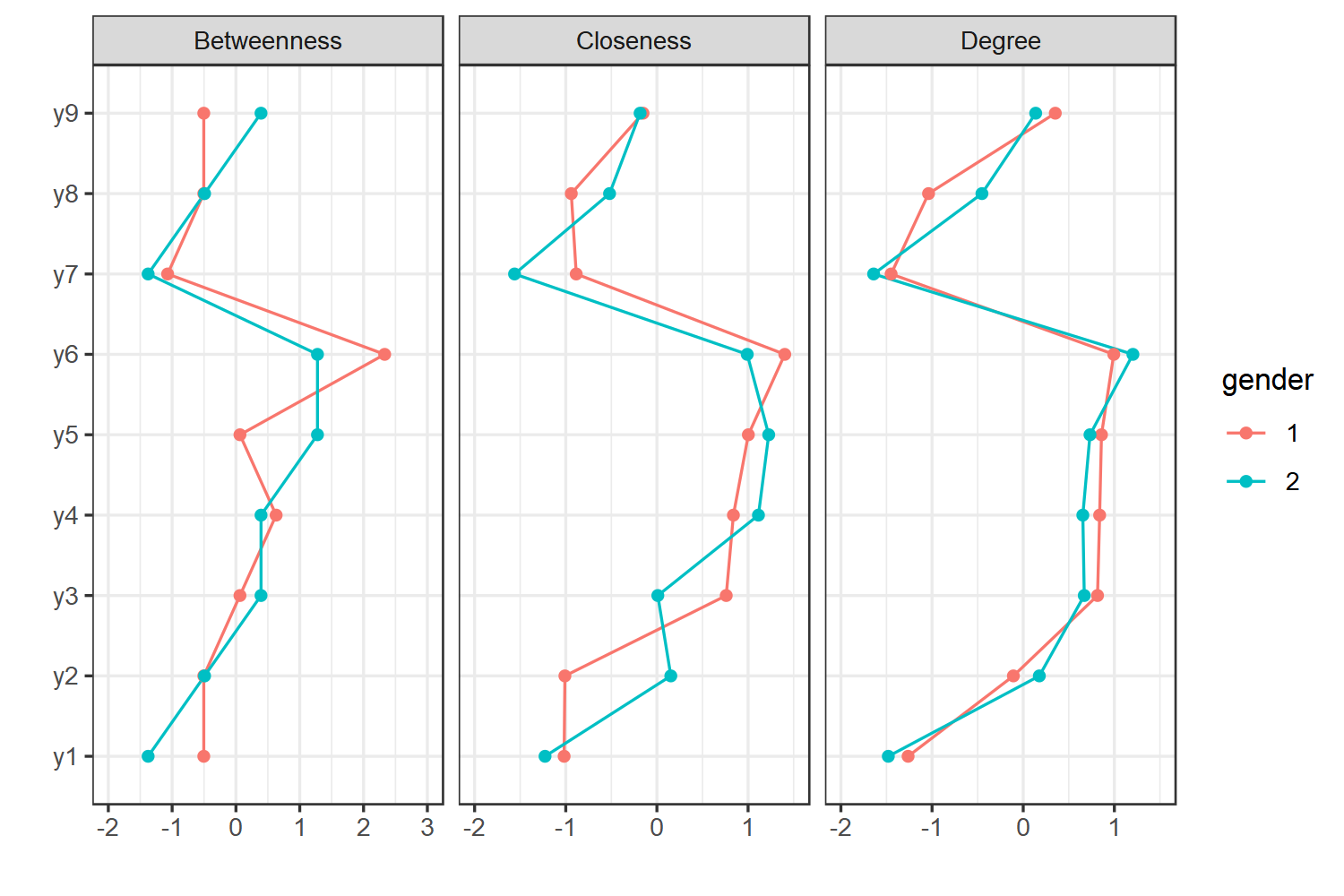


**Appendix S11.** Standardized estimates of node centrality in the network between males (1) and females (2).


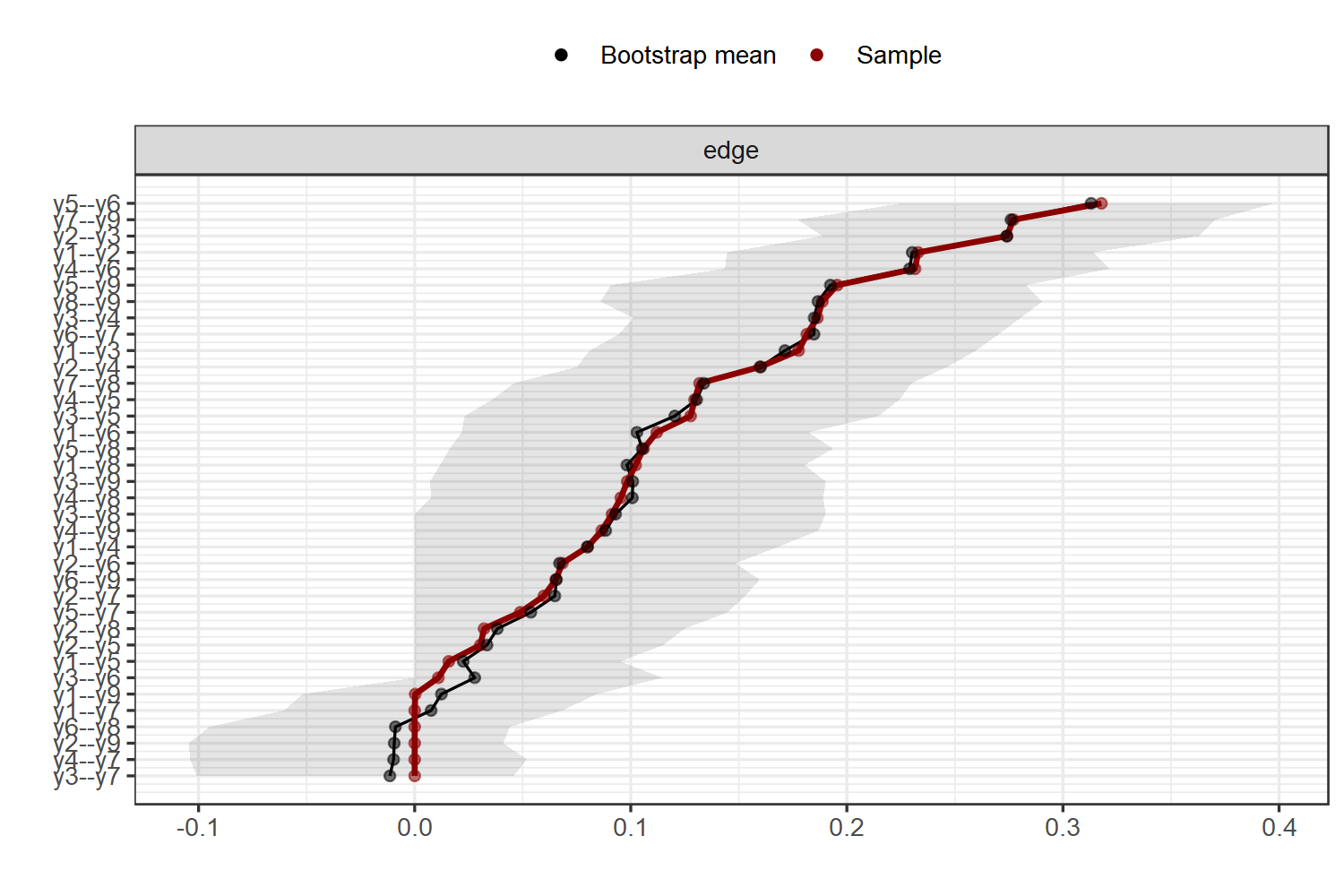


**Appendix S12.** Edge stability in the network among 928 males


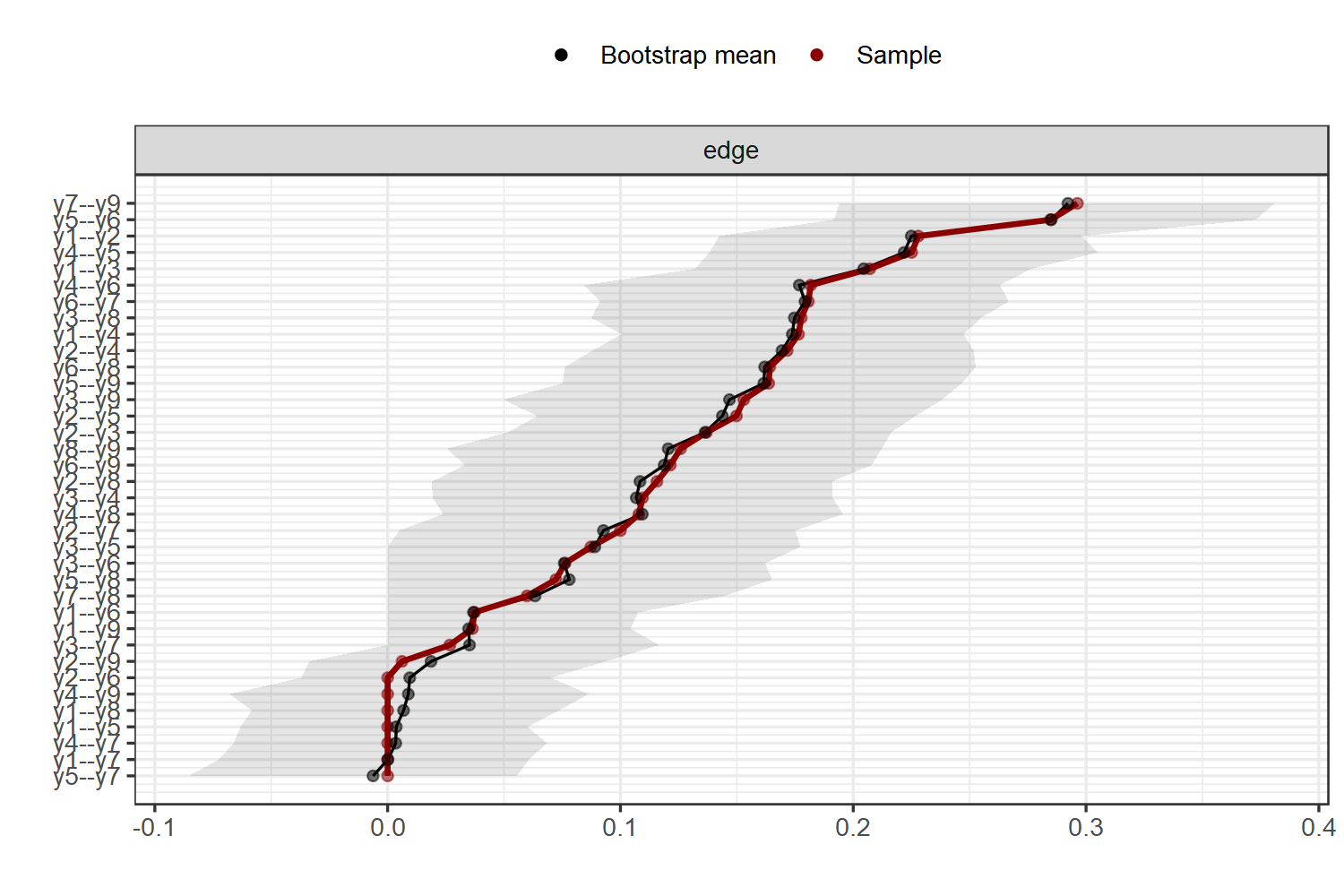


**Appendix S13.** Edge stability in the network among 957 females


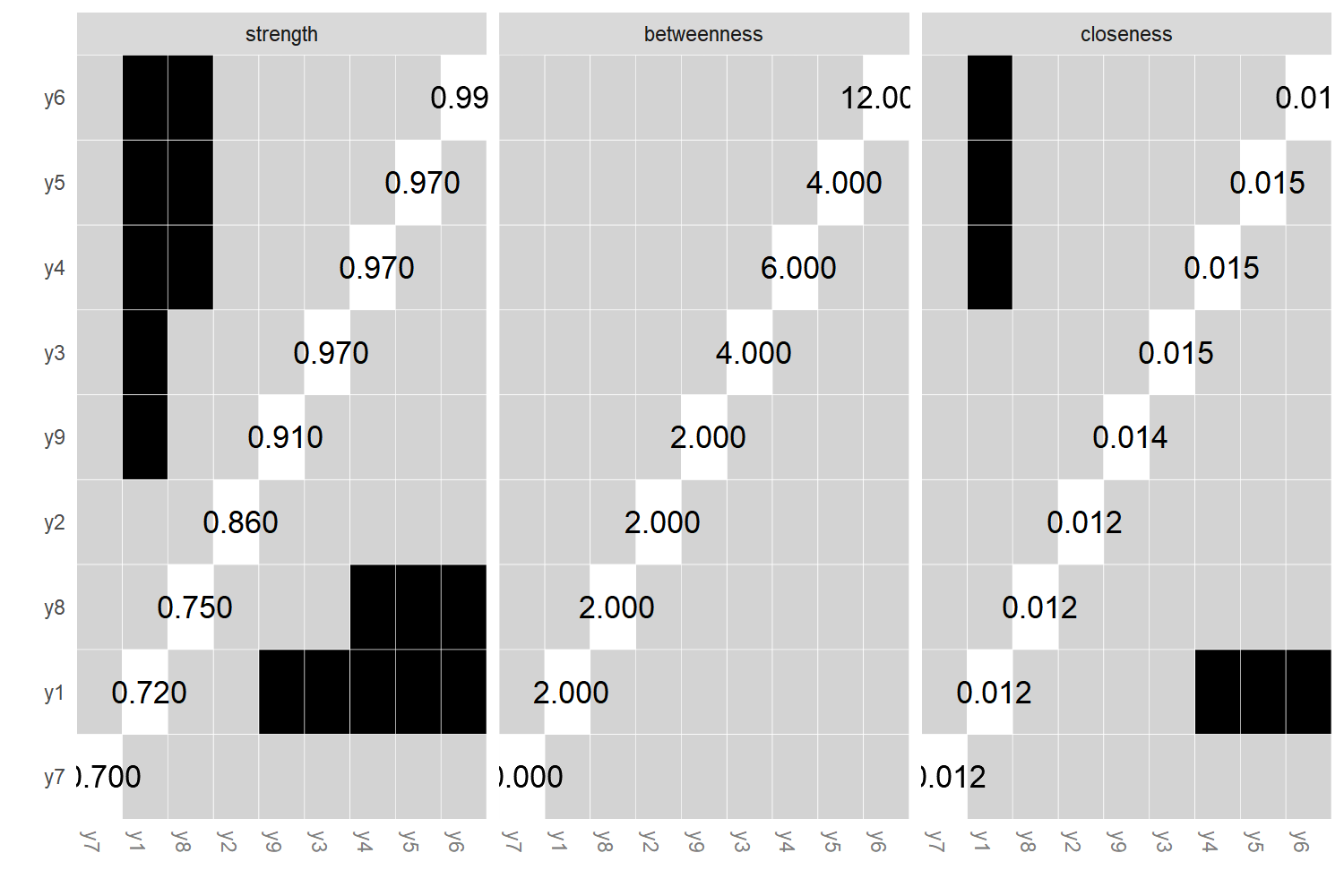


**Appendix S14.** Centrality stability in the network among 928 males


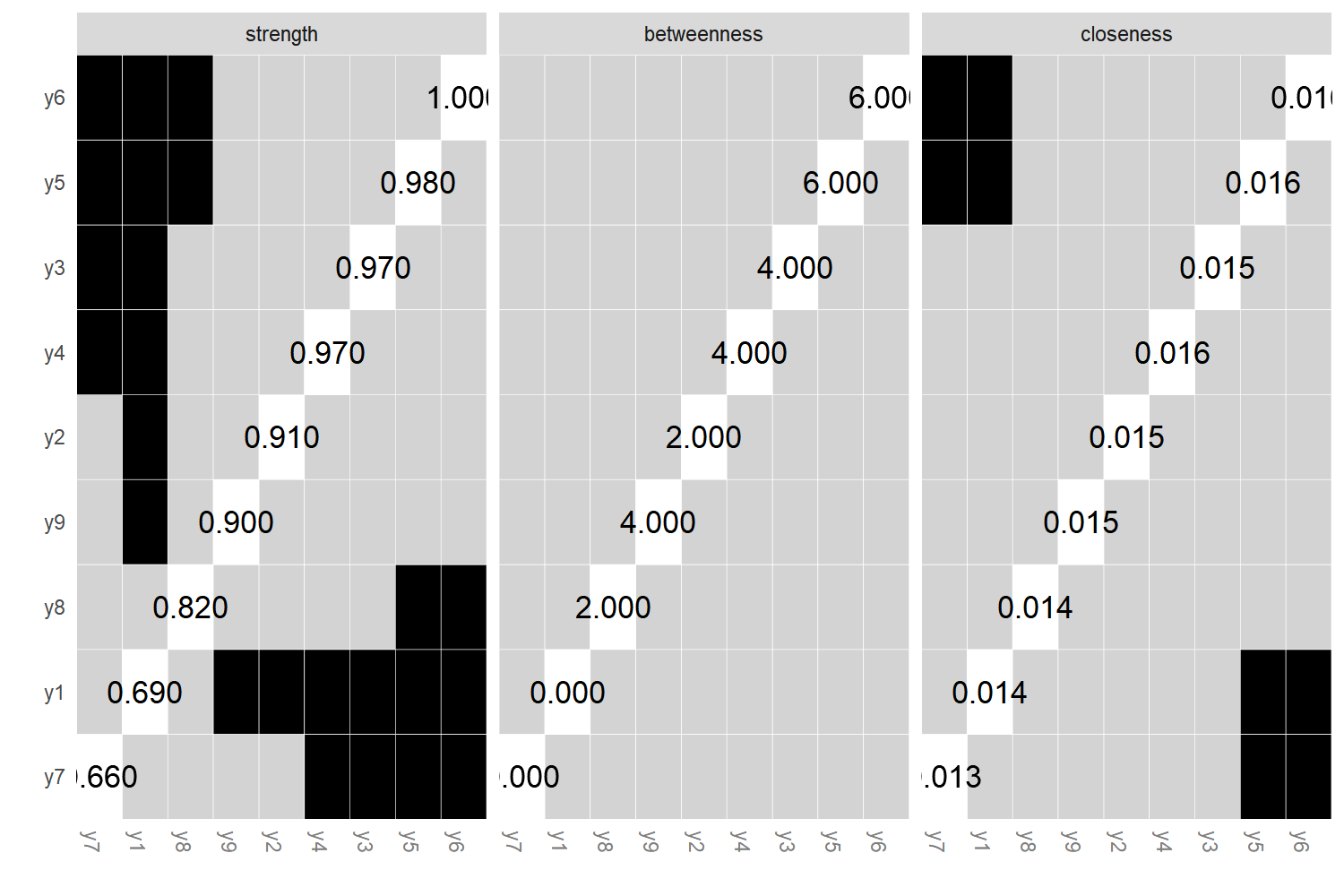


**Appendix S15.** Centrality stability in the network among 957 females

**Appendix S16 Edge weight matrix of the facet-level network among 533 Bangladesh participants**

| Variables | y1 | y2 | y3 | y4 | y5 | y6 | y7 | y8 | y9 |
| --- | --- | --- | --- | --- | --- | --- | --- | --- | --- |
| y1 | 0.000 | 0.233 | 0.162 | 0.150 | 0.038 | 0.000 | 0.000 | 0.053 | 0.000 |
| y2 | 0.233 | 0.000 | 0.221 | 0.295 | 0.214 | 0.017 | 0.053 | 0.068 | 0.043 |
| y3 | 0.162 | 0.221 | 0.000 | 0.121 | 0.071 | 0.105 | 0.000 | 0.181 | 0.010 |
| y4 | 0.150 | 0.295 | 0.121 | 0.000 | 0.024 | 0.172 | 0.000 | 0.044 | 0.000 |
| y5 | 0.038 | 0.214 | 0.071 | 0.024 | 0.000 | 0.351 | 0.000 | 0.000 | 0.266 |
| y6 | 0.000 | 0.017 | 0.105 | 0.172 | 0.351 | 0.000 | 0.245 | 0.106 | 0.067 |
| y7 | 0.000 | 0.053 | 0.000 | 0.000 | 0.000 | 0.245 | 0.000 | 0.027 | 0.348 |
| y8 | 0.053 | 0.068 | 0.181 | 0.044 | 0.000 | 0.106 | 0.027 | 0.000 | 0.210 |
| y9 | 0.000 | 0.043 | 0.010 | 0.000 | 0.266 | 0.067 | 0.348 | 0.210 | 0.000 |

**Appendix S17 Edge weight matrix of the facet-level network among 702 Iran participants**

| Variables | y1 | y2 | y3 | y4 | y5 | y6 | y7 | y8 | y9 |
| --- | --- | --- | --- | --- | --- | --- | --- | --- | --- |
| y1 | 0.000 | 0.219 | 0.175 | 0.149 | 0.032 | 0.120 | 0.000 | 0.010 | 0.099 |
| y2 | 0.219 | 0.000 | 0.230 | 0.177 | 0.131 | 0.042 | 0.066 | 0.203 | 0.000 |
| y3 | 0.175 | 0.230 | 0.000 | 0.047 | 0.058 | 0.017 | 0.136 | 0.032 | 0.000 |
| y4 | 0.149 | 0.177 | 0.047 | 0.000 | 0.135 | 0.197 | -0.080 | 0.013 | 0.171 |
| y5 | 0.032 | 0.131 | 0.058 | 0.135 | 0.000 | 0.172 | 0.095 | 0.089 | 0.108 |
| y6 | 0.120 | 0.042 | 0.017 | 0.197 | 0.172 | 0.000 | 0.012 | 0.108 | 0.112 |
| y7 | 0.000 | 0.066 | 0.136 | -0.080 | 0.095 | 0.012 | 0.000 | 0.121 | 0.315 |
| y8 | 0.010 | 0.203 | 0.032 | 0.013 | 0.089 | 0.108 | 0.121 | 0.000 | 0.010 |
| y9 | 0.099 | 0.000 | 0.000 | 0.171 | 0.108 | 0.112 | 0.315 | 0.010 | 0.000 |

**Appendix S18 Edge weight matrix of the facet-level network among 666 Pakistan participants**

| Variables | y1 | y2 | y3 | y4 | y5 | y6 | y7 | y8 | y9 |
| --- | --- | --- | --- | --- | --- | --- | --- | --- | --- |
| y1 | 0.000 | 0.168 | 0.175 | 0.114 | 0.023 | 0.091 | 0.028 | 0.201 | 0.012 |
| y2 | 0.168 | 0.000 | 0.374 | 0.206 | 0.059 | 0.013 | 0.171 | 0.049 | 0.031 |
| y3 | 0.175 | 0.374 | 0.000 | 0.126 | 0.025 | 0.081 | 0.000 | 0.079 | 0.027 |
| y4 | 0.114 | 0.206 | 0.126 | 0.000 | 0.201 | 0.246 | -0.095 | 0.151 | 0.000 |
| y5 | 0.023 | 0.059 | 0.025 | 0.201 | 0.000 | 0.366 | -0.050 | 0.085 | 0.116 |
| y6 | 0.091 | 0.013 | 0.081 | 0.246 | 0.366 | 0.000 | 0.243 | 0.005 | 0.079 |
| y7 | 0.028 | 0.171 | 0.000 | -0.095 | -0.050 | 0.243 | 0.000 | 0.060 | 0.302 |
| y8 | 0.201 | 0.049 | 0.079 | 0.151 | 0.085 | 0.005 | 0.060 | 0.000 | 0.165 |
| y9 | 0.012 | 0.031 | 0.027 | 0.000 | 0.116 | 0.079 | 0.302 | 0.165 | 0.000 |

Appendix S19 Centrality measures per variable of the network among three countries

| Variables | Bangladesh | | | Iran | | | Pakistan | | |
| --- | --- | --- | --- | --- | --- | --- | --- | --- | --- |
|  | Betweenness | Closeness | Strength | Betweenness | Closeness | Strength | Betweenness | Closeness | Strength |
| y1 | -0.904 | -1.422 | -1.279 | -0.356 | -0.008 | -0.098 | -0.278 | -1.008 | -0.842 |
| y2 | 1.622 | 1.310 | 1.551 | 2.316 | 1.609 | 1.783 | 1.390 | 1.013 | 0.905 |
| y3 | -0.062 | -0.318 | 0.029 | 0.178 | -0.459 | -0.888 | -1.112 | -0.960 | -0.338 |
| y4 | -0.904 | -0.382 | -0.334 | 0.178 | 1.777 | 1.091 | 1.390 | 1.348 | 1.364 |
| y5 | 1.341 | 1.463 | 0.544 | -0.891 | -0.840 | 0.009 | -1.112 | -0.816 | -0.084 |
| y6 | -0.624 | 0.749 | 1.108 | -0.891 | -0.591 | -0.272 | 0.556 | 0.594 | 1.274 |
| y7 | -0.904 | -0.848 | -1.075 | 0.178 | -0.762 | 0.050 | 0.556 | 1.141 | 0.073 |
| y8 | -0.343 | -0.746 | -0.985 | -0.891 | -0.634 | -1.660 | -0.695 | -0.790 | -0.962 |
| y9 | 0.780 | 0.195 | 0.441 | 0.178 | -0.091 | -0.014 | -0.695 | -0.523 | -1.391 |


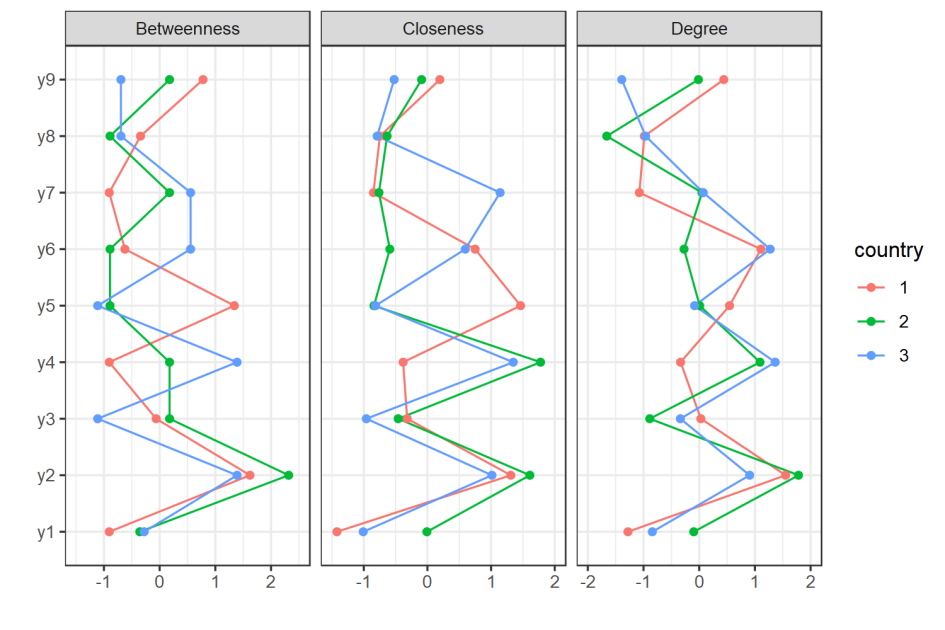


**Appendix S20.** Standardized estimates of node centrality in the network among Bangladesh (1), Iran (2), and Pakistan (3).


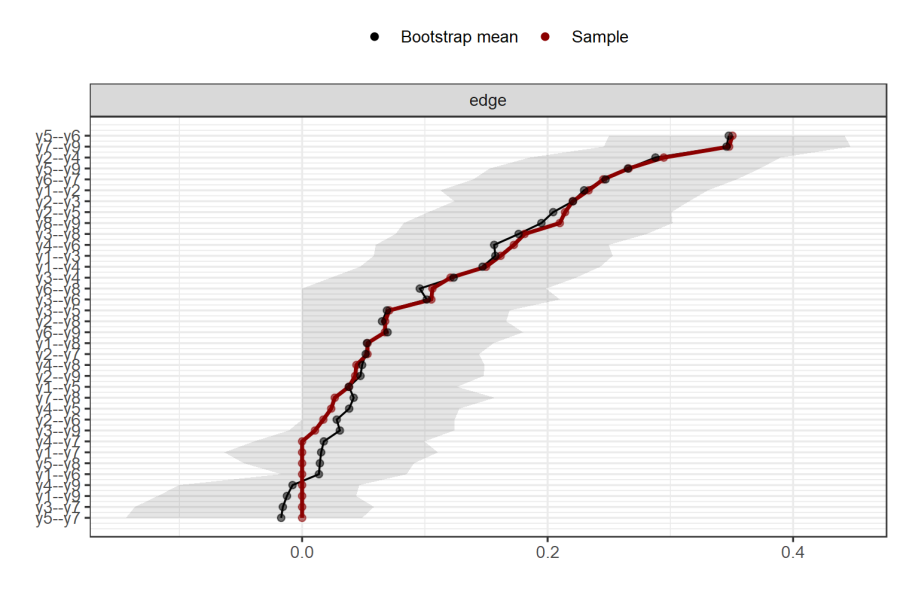


**Appendix S21.** Edge stability in the network among 533 Bangladesh participants


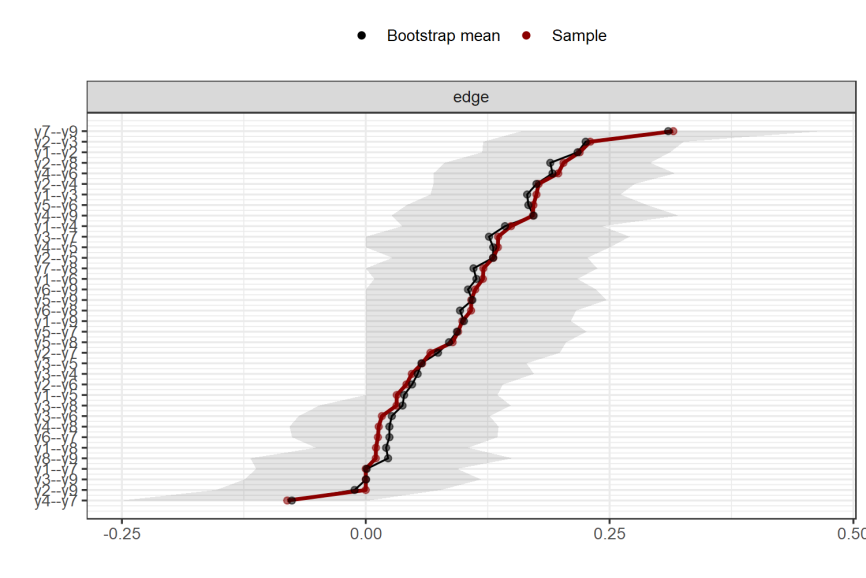


**Appendix S22.** Edge stability in the network among 702 Iran participants


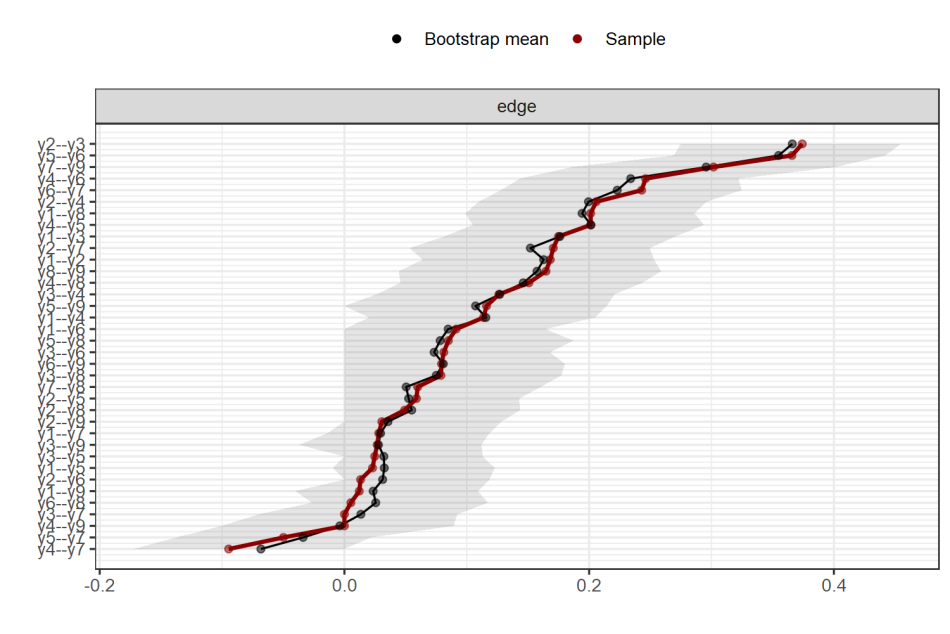


**Appendix S23.** Edge stability in the network among 666 Pakistan participants


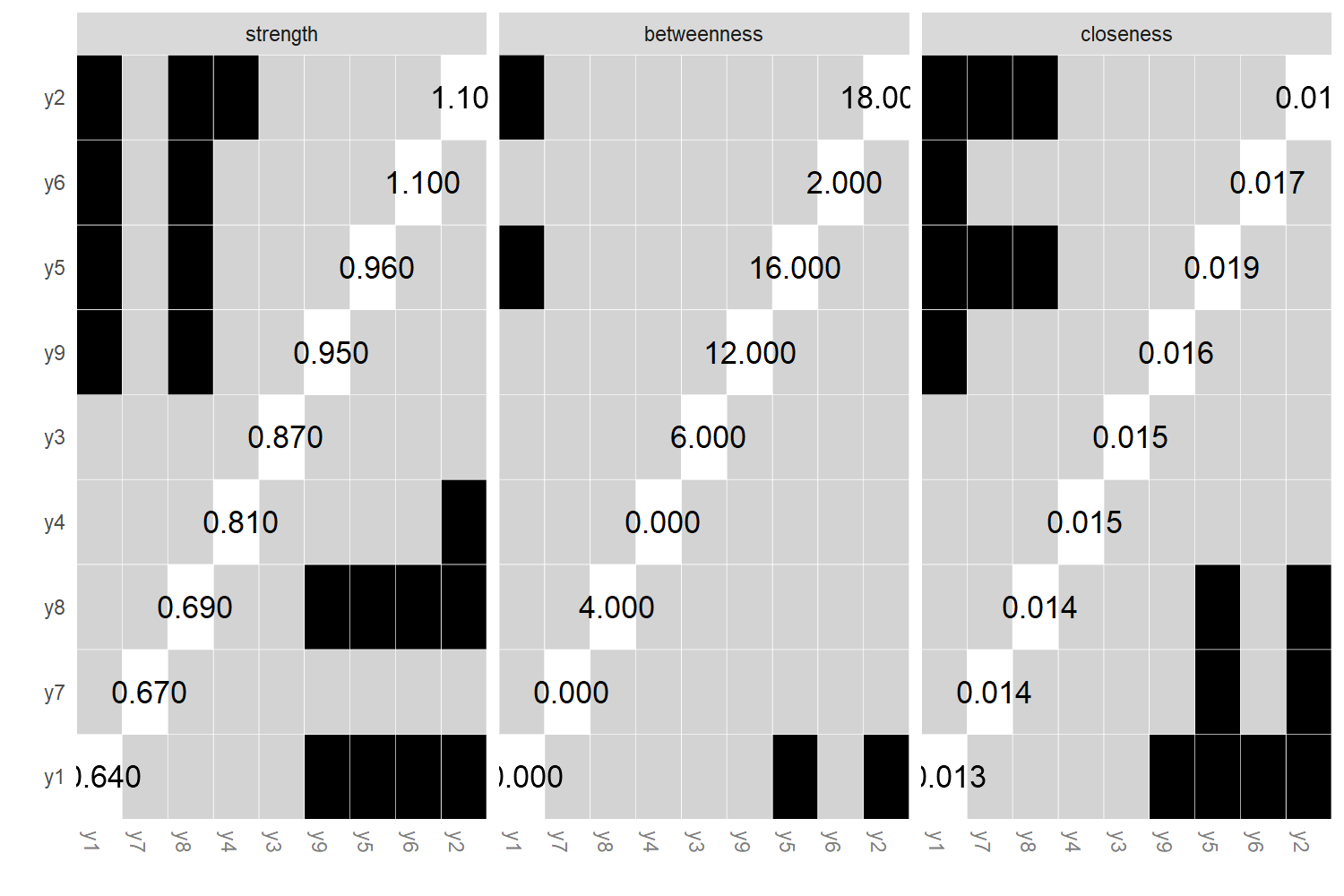


**Appendix S24.** Centrality stability in the network among 533 Bangladesh participants


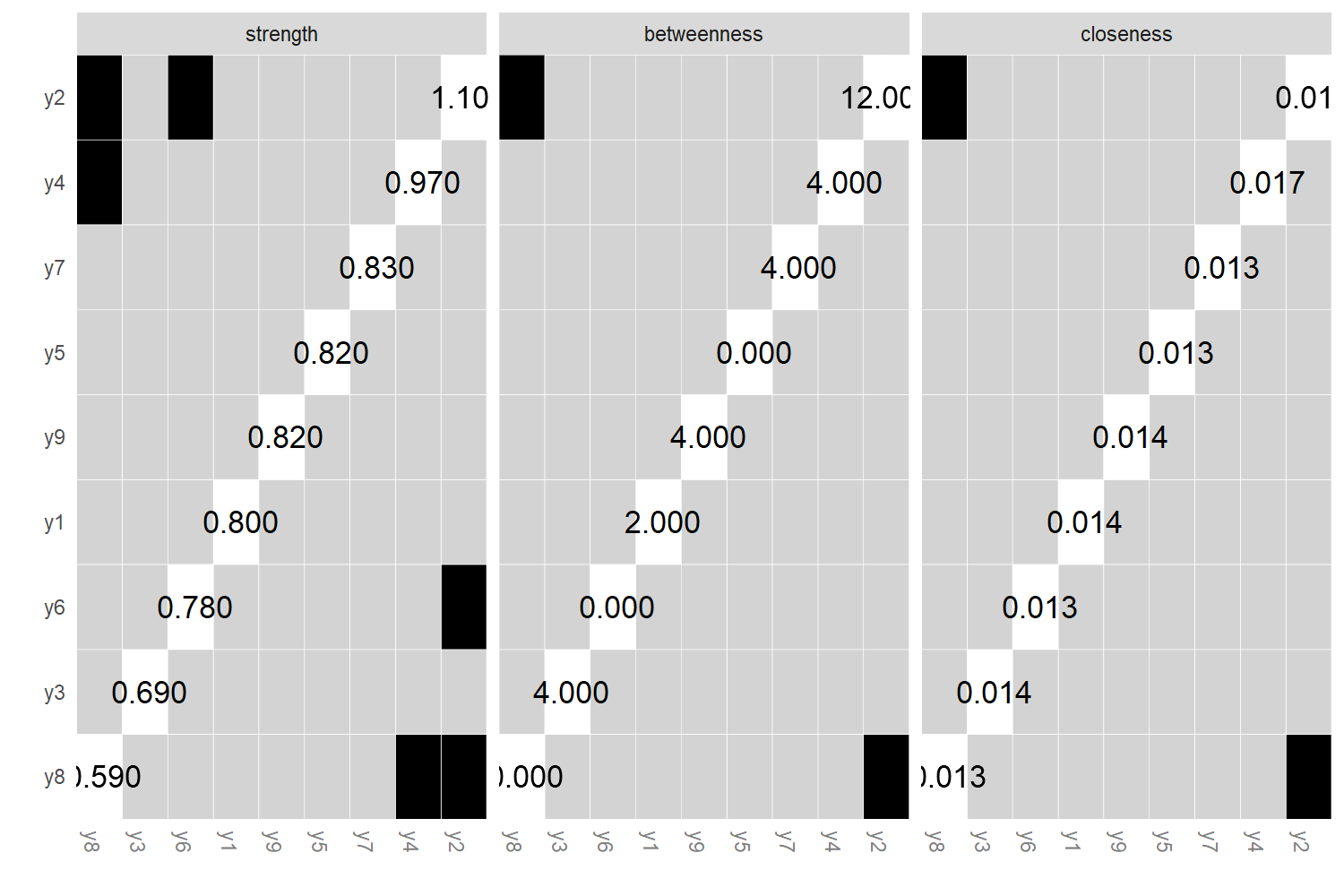


**Appendix S25.** Centrality stability in the network among 702 Iran participants


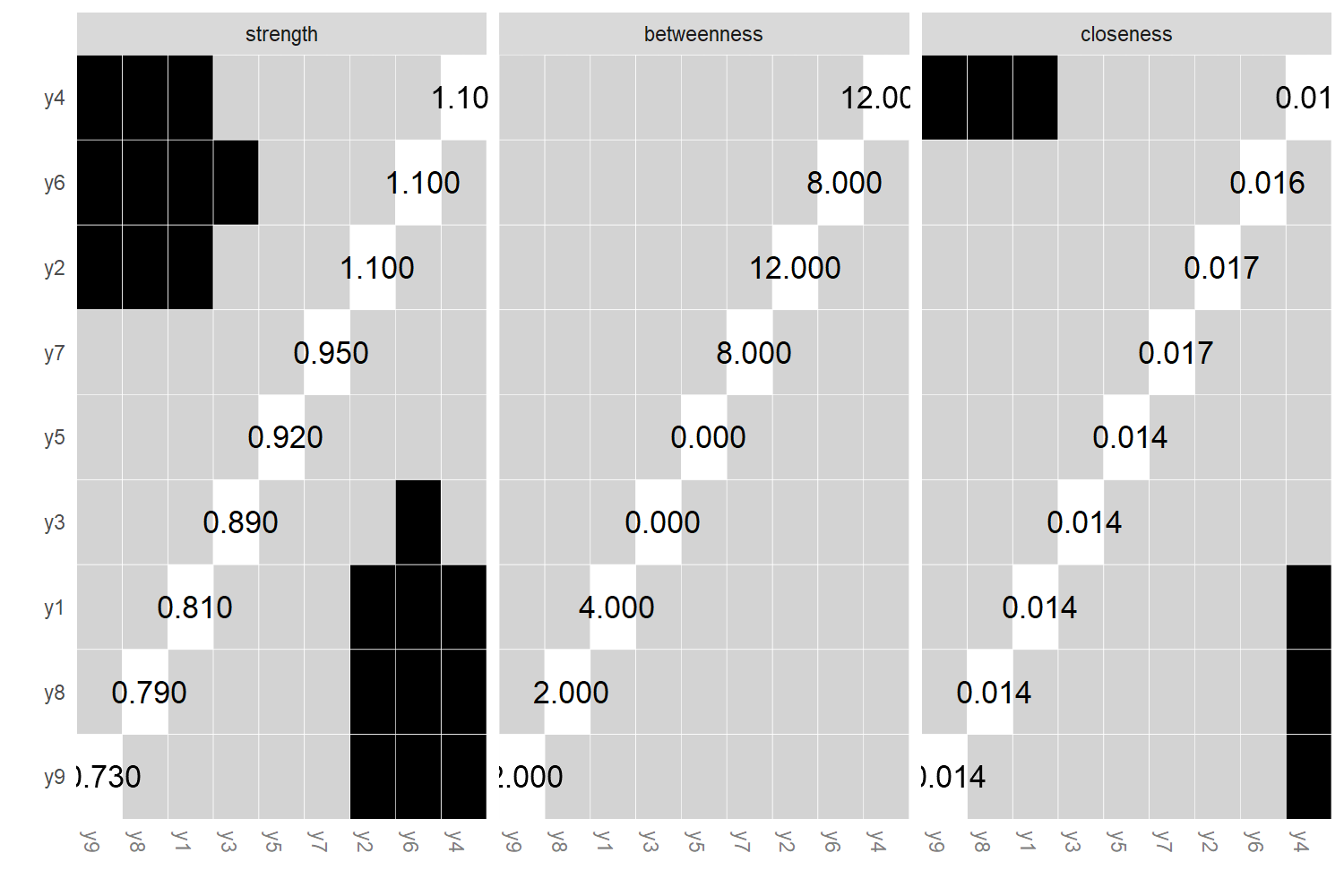


**Appendix S26.** Centrality stability in the network among 666 Pakistan participants
